# Alpha-pinene ameliorates liver fibrosis by suppressing oxidative stress, inflammation, and the TGF-β/Smad3 signaling pathway

**DOI:** 10.22038/ijbms.2025.81693.17678

**Published:** 2025

**Authors:** Fatemeh Noroozi, Masoumeh Asle-Rousta, Rahim Amini, Zeinab Sahraeian

**Affiliations:** 1 Department of Physiology, Zanjan Branch, Islamic Azad University, Zanjan, Iran; 2 Department of Biology, Zanjan Branch, Islamic Azad University, Zanjan, Iran; 3 Nanobiotechnology Research Center, Zanjan Branch, Islamic Azad University, Zanjan, Iran

**Keywords:** Alpha-pinene, Carbon tetrachloride, Collagen, Glutathione, Inflammation, Toll-like receptor 4

## Abstract

**Objective(s):**

A monoterpene alpha-pinene possesses anti-oxidant, anti-inflammatory, and anti-apoptotic properties. Here, we investigated the effect of alpha-pinene on molecular, biochemical, and histological changes induced by carbon tetrachloride (CCl_4_) in the liver of male Wistar rats.

**Materials and Methods:**

Animals were divided into four groups: Control, Pinene, CCl_4_, and CCl_4_.Pinene. Pinene and CCl_4_.Pinene groups were given alpha-pinene (50 mg/kg/day) through intraperitoneal (IP) injections for six consecutive weeks. CCl_4_ and CCl_4_.Pinene groups received IP injections of CCl_4_ (2 ml/kg twice weekly for six consecutive weeks).

**Results:**

The results revealed that alpha-pinene inhibited enhancing liver enzyme AST (*P*<0.001), ALT (*P*<0.001), ALP (*P*<0.01), and GGT (*P*<0.001) activity in CCl_4_.Pinene rats. It reduced malondialdehyde (*P*<0.05) and nitric oxide (*P*<0.05) levels and increased the catalase enzyme activity (*P*<0.05) and glutathione levels (*P*<0.01) in the liver. Likewise, alpha-pinene suppressed proinflammatory and profibrotic gene expression and prevented significant histological damage and collagen deposition in the liver of these animals. Also, alpha-pinene reduced the expression of TLR4 (*P*<0.01), NF-κB (*P*<0.05), PI3K (*P*<0.05), Akt (*P*<0.05), mTOR (*P*<0.01), TGF-β1 (*P*<0.01), and Smad3 (*P*<0.01) in the liver of rats receiving CCl_4_.

**Conclusion:**

We concluded that alpha-pinene reduced CCl_4_-induced liver fibrosis by lowering oxidative stress, suppressing liver inflammation, and inhibiting TLR4/NF-κB, TGF-β/Smad3, and PI3K/Akt/mTOR signaling pathways. Consequently, alpha-pinene may have potential therapeutic value in treating liver diseases.

## Introduction

Carbon tetrachloride (CCl_4_) is a highly toxic substance that has been widely used in various studies to induce liver fibrosis and cirrhosis. Additionally, evidence shows its harmful effects on other organs such as the kidneys, testicles, and brain. CCl_4_ metabolites in the body promote lipid peroxidation, leading to damage to proteins and DNA. By increasing the production of oxidation products like protein carbonyls and malondialdehyde (MDA), CCl_4_ hinders protein production and function, including membrane proteins. This can destroy the cell membrane. As a consequence of the loss of integrity of the liver cell membrane, liver enzymes such as alanine transaminase (ALT), aspartate transaminase (AST), alkaline phosphatase (ALP), and gamma-glutamyl transferase (GGT) leak into the plasma (1, 2).

CCl_4_ weakens the body’s anti-oxidant system by reducing the activity of enzymes such as catalase, superoxide dismutase, and glutathione peroxidase, as well as lowering glutathione levels. It also leads to increased lipid peroxidation and nitric oxide production in the liver. This can cause oxidative and nitrosative stress, which can damage liver cells (1, 3, 4). CCl_4_ triggers an inflammatory response in the liver by increasing the production of inflammatory factors such as tumor necrosis factor (TNF), interleukin (IL)-1β, and IL-6, which are essential to liver fibrosis (2, 5). Research indicates that there is a direct relationship between increased oxidative stress and inflammatory factors and the development of nonalcoholic fatty liver disease (NAFLD). If NAFLD is not prevented, it can progress to nonalcoholic steatohepatitis and eventually to fibrosis, cirrhosis, and hepatocarcinoma (6). Additionally, CCl_4_ can contribute to the emergence and worsening of NAFLD and steatohepatitis by intensifying oxidative stress and inflammation in the liver (7).

Transforming growth factor (TGF)-β is produced by stellate cells, Kupffer cells, and hepatocytes in response to CCl_4_. TGF-β plays a crucial role in promoting liver fibrosis by reducing extracellular matrix remodeling through metalloproteinase-2 (MMP-2) production. It also stimulates liver collagen type I (Col-I) production through Smads and non-Smad signaling pathways (8-11). Research on fibrosis treatment is focused on suppressing oxidative stress and inflammation, as well as inhibiting signaling pathways such as phosphoinositide 3-kinase (PI3K)/anti-apoptotic kinase (Akt)/mammalian target of rapamycin (mTOR), Toll-like receptor (TLR) 4/nuclear factor kappa-light-chain-enhancer of activated B cells (NF-κB), and TGF-ß 1/Smad3. These signaling pathways play an important role in the development and progression of fibrosis (12-15). Natural compounds make a significant contribution to this field of research (16). 

Alpha-pinene ((1RS,5RS)-2,6,6-trimethylbicyclo[3.1.1]hept-2-ene) is a monoterpene found in coniferous trees and plants such as Piper nigrum and Cannabis sativa L. This compound has a wide range of pharmacological effects (17). Alpha-pinene is anti-oxidant, anti-inflammatory, anti-apoptotic, anti-diabetic, cardioprotective, and neuroprotective (18-22). Despite this, few reports have been published on its potential for hepatoprotection. According to Santos *et al.* (21), alpha-pinene reduces liver enzyme levels of AST and ALT in diabetic rats. Moreover, alpha-pinene-containing plants like Myrtus communis L. and Rosmarinus officinalis L. are hepatoprotective (23, 24). Based on these findings, the hypothesis was proposed that alpha-pinene could also protect the liver. In the current study, alpha-pinene was examined in adult male Wistar rats to determine whether it affects oxidative and nitrosative stress, inflammation, liver enzyme levels, Col-I and MMP2 expression, and histological changes as a result of CCl_4_ treatment. We also investigated the possible mechanisms for hepatoprotection of alpha-pinene by studying the TLR4/NF-κB, TGF-β/Smad2/3, and PI3K/Akt/mTOR signaling pathways.

## Materials and Methods

### Material

Alpha-pinene, ketamine, xylazine, dimethyl sulfoxide (DMSO), and 5, 5′-dithiobis-(2-nitrobenzoic acid) (DTNB) were obtained from Sigma-Aldrich (USA). All primers were purchased from Bioneer (Korea). The kits for biochemical and molecular studies are introduced in the relevant sections. CCl_4_ and other chemicals were purchased from Merck (Germany). 

### Animals and research design

Twenty-four male Wistar rats weighing 200–220 g were purchased from Shahid Beheshti University of Medical Sciences (Iran). Throughout the experiment, rats were maintained under standard conditions (12/12 hr of light-dark cycle, temperature 23–25 °C, and easy access to food and water) at the Nanobiotechnology Research Center of Islamic Azad University, Zanjan Branch. 

**Figure 1:**
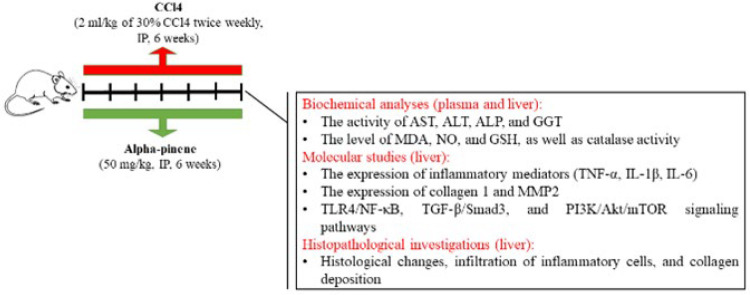
Research timeline

Animals were placed in cages in groups of four to habituate themselves to the laboratory conditions. One week later, the rats were divided into four groups of six. The Control group did not receive any treatment (C). Alpha-pinene (50 mg/kg diluted in DMSO) was administered intraperitoneally for six consecutive weeks to the Pinene group based on its neuroprotective effect in a model of Alzheimer’s disease (20). During the same period, the CCl_4_ group received 2 ml/kg of 30% CCl_4_ twice weekly (intraperitoneally) (25). The CCl4.Pinene group was also treated with both substances. At the end of the six weeks, animals were sacrificed under ketamine (50 mg/kg)-xylazine (10 mg/kg) anesthesia (26). Next, biochemical, molecular, and histological analyses were conducted on blood and liver samples. For biochemical and histological investigations, all animals were sampled. For molecular studies, 5 rats from each group were used. Figure 1 shows the research timeline.

The Animal Ethics Committee of Islamic Azad University, Zanjan branch approved the study (Code: IR.IAU.Z.REC.1401.036).

### Assessment of liver enzyme activity

According to the instructions, we measured the AST, ALT, ALP, and GGT enzyme levels in serum using Bionik enzyme kits (Bionik, Iran).

### Liver homogenization for biochemical studies

Liver samples were homogenized in Tris-HCl buffer (pH 7.5, 0.25 M) and centrifuged (12000 g, 20 min, 4 °C). Supernatant protein concentrations were determined using the Lowry method (27). 

### Lipid peroxidation assay

MDA levels in the liver were measured to determine lipid peroxidation. A pink color is produced when MDA reacts with thiobarbituric acid (TBA). For measuring MDA, 250 μl of homogenized liver tissue was mixed with 500 μl of trichloroacetic acid (TCA) and heated at 95 °C for 15 min. Then the samples were centrifuged (14000 g, 5 min). 250 μl of TBA solution was added to the supernatant and placed in a hot water bath at 95 °C for 10 min, and its absorbance was read at 532 nm wavelength. The concentration of MDA was expressed in nmol/mg protein (28).

### Measurement of nitric oxide

The amount of nitrite, which is one of the products of nitric oxide, was measured using a kit purchased from ArsamFaraZist (Iran). Under acidic conditions, NO2^-^ reacts with sulfanilamide and N-(1-Naphthyl) ethylenediamine dihydrochloride (NED) to form the azo compound, which appears pink (Griess reaction). 20 μl of liver homogenate was mixed with 880 μl of distilled water. The next step was to add 50 μl of sulfonamide and incubate it for 5 min at room temperature. Afterward, 50 μl of NED reagent was added, and its absorbance was measured at 520 nm. NO concentration was expressed in nmol/mg protein (20).

### GSH evaluation

This test measures glutathione by detecting the reaction between its thiol and the DTNB. GSH levels were evaluated by mixing homogenized tissue (100 μl) with diluent buffer (to a volume of 400 μl). Afterward, 100 μl of sulfosalicylic acid was added and incubated on ice for 10 min before centrifugation (12,000 g, 5 min). A yellow color was created by adding 400 μl of reaction buffer and 100 μl of DTNB to the supernatant. As a final step, its absorption was read at 412 nm. The amount of GSH was expressed as nmol/mg of protein (20).

### Determination of catalase activity

The activity of catalase was evaluated by its peroxidase function. As a result of the reaction of the catalase enzyme with methanol, formaldehyde is produced. Chromogen reagent and formaldehyde form a heterocyclic ring that changes color from colorless to purple during oxidation. A mixture of 200 µl of reaction buffer, 150 µl of methanol, and 30 µl of H_2_0 was gently shaken. It was then mixed with 50 µl of homogenate and then incubated in the dark for 20 min. The potassium hydroxide solution and chromogen reagent were then added and incubated for 10 min. Afterward, 150 µl of potassium periodate was added to the samples and centrifuged (10,000 g, 10 min). At 550 nm, the absorbance is measured, and the catalase activity is expressed as U/mg of protein (20).

### Evaluation of mRNA expression

The expression levels of TNF-α, IL-1β, IL-6, NF-κB, TLR4, TGF-B, Smad2, Smad3, PI3K, Akt, mTOR, Col-1, and MMP2 in the liver were determined by real-time PCR. A Parstous RNA isolation kit (Iran) was used to isolate total RNA from frozen tissues. Based on the A260/A280 ratio and spectrophotometric measurements at 260 nm, RNA concentration, and quality were determined. A total of 1µg of RNA from each sample was reverse transcribed with the Easy cDNA Synthesis Kit (Parstous, Iran). An ABI StepOnePlus thermocycler (Applied Biosystems, USA) was used to conduct real-time polymerase chain reactions (PCR) using RealQ Plus 2x Master Mix Green High RoxTM (Ampliqon, Denmark). Initial activation was carried out at 95 °C for 15 min, followed by 40 cycles of denaturation at 95 °C for 20 sec and annealing/extension at 60 °C for 60 sec. To validate the single PCR product of each primer, melting curves were analyzed. Glyceraldehyde-3-phosphate dehydrogenase (GAPDH) was used as the housekeeping gene and the relative expression of genes was calculated based on the 2^-ΔΔCT^ comparative expression method (29). The sequences of primers are listed in Table 1. 

### Histological investigation

The liver tissue was removed, fixed with 10% formalin, and then embedded in paraffin wax. Sections with a thickness of 5 µm were prepared and stained with hematoxylin-eosin (H&E) and Masson’s trichrome. Histopathological changes were studied using a light microscope. To determine the percentage of inflamed areas in the liver, ten cross-sections of each animal’s liver were examined in sections stained with H&E. Ten fields of liver from each animal were also investigated in sections stained with Masson’s trichrome to define evidence of fibrosis using Image J software (30).

### Statistical analyses

We analyzed the data using SPSS version 16.0 software. The results were presented as the mean ± standard error of the mean (SEM). The differences among groups were detected by one-way ANOVA followed by the Tukey LSD test for *post hoc* analysis. Statistical comparison of fibrotic areas and inflamed areas between CCl_4_ and CCl_4_.Pinene groups were performed by t-test. It was considered statistically significant if the *P-*value was less than 0.05.

## Results

### The effect of alpha-pinene on the level of liver enzymes

The injection of CCl_4_ resulted in a significant increase in the levels of AST, ALT, ALP, and GGT when compared to the Control group (*P*=0.0001), However, in the CCl_4_.Pinene group compared to the CCl_4_ group, there was a significant decrease in the levels of these enzymes (*P*=0.0001, *P*=0.0001, *P*=0.004, and *P*=0.0001, respectively). The activity of liver enzymes in the Pinene-group animals was not significantly different from the Control group (Table 2).

### Effect of alpha-pinene on oxidative/nitrosative stress

**Figure 2:**
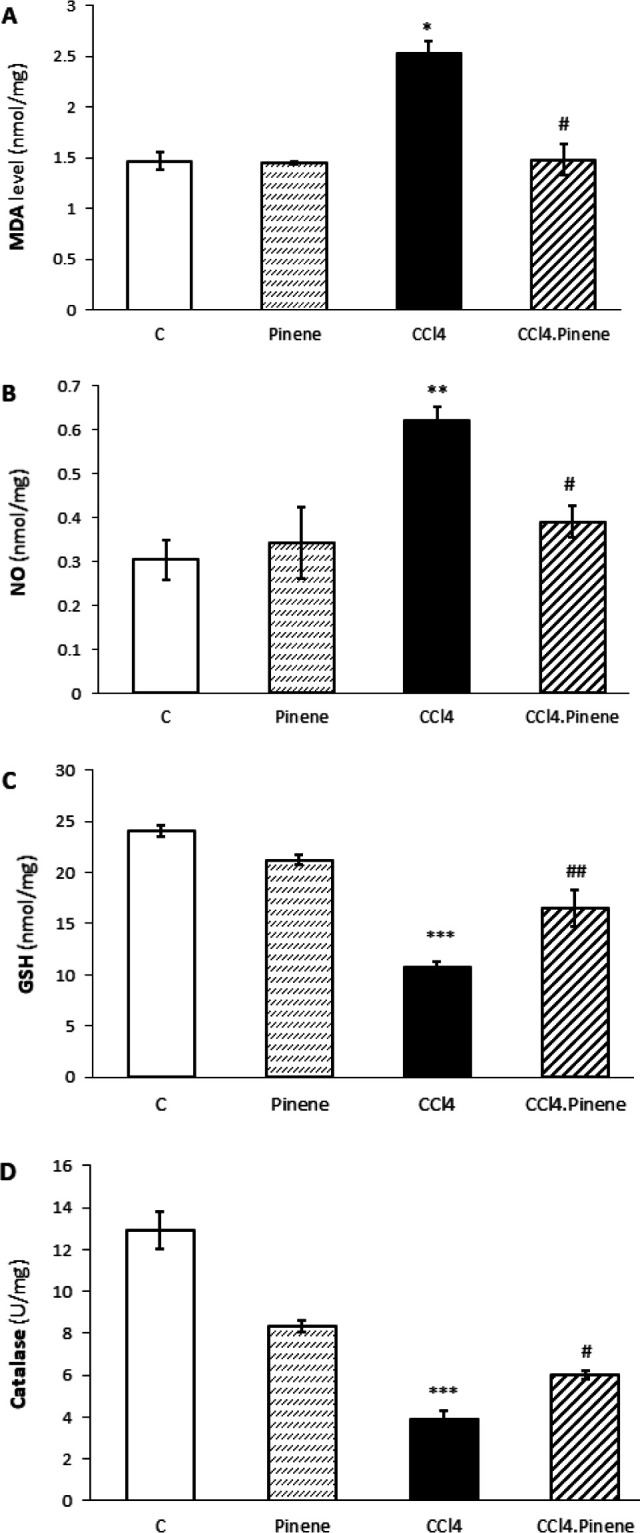
Effect of alpha-pinene on hepatic levels of (A) malondialdehyde (MDA), (B) nitric oxide (NO), (C) reduced glutathione (GSH), and (D) activity of catalase enzyme in CCl_4_-injected rats

CCl_4_ increased MDA (*P*=0.038) and NO (*P*=0.006) levels in liver tissue in comparison to the Control group. However, alpha-pinene decreased both factors in the CCl_4_.Pinene group when compared to the CCl_4_ group (*P*=0.023). Additionally, CCl_4_ significantly reduced catalase activity GSH content in rats’ livers (*P*=0.0001). In the CCl_4_.Pinene group, daily treatment with alpha-pinene significantly increased catalase activity (*P*=0.030) and GSH levels (*P*=0.004). It is worth mentioning that there were no significant differences between the Pinene and Control groups in any of the factors studied here, as shown in Figure 2.

### Effect of alpha-pinene on the expression of proinflammatory factors

**Figure 3:**
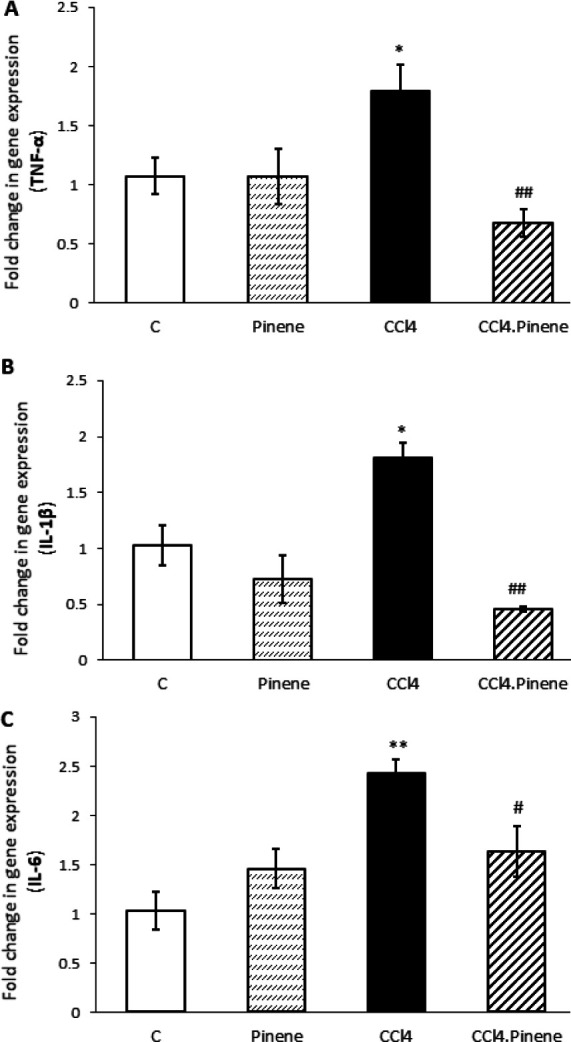
Effect of alpha-pinene on (A) TNF-α, (B) IL-1β, and (C) IL-6 mRNA expression in the liver of CCl_4_-injected rats

Compared to the Control group, animals exposed to CCl_4 _showed significant increases in mRNA levels of TNF-α, IL-1β, and IL-6 (*P*=0.034, *P*=0.020, and *P*=0.001, respectively). However, injection of alpha-pinene significantly decreased the expression of TNF-α (*P*=0.005), IL-1β (*P*=0.003), and IL-6 (*P*=0.024) in animals receiving CCl_4_. The animals in the Pinene group did not show significantly different levels of proinflammatory factors in their livers when compared to the Control group (Figure 3).

### Effect of alpha-pinene on the expression of MMP-2 and Col-1 in the liver

**Figure 4:**
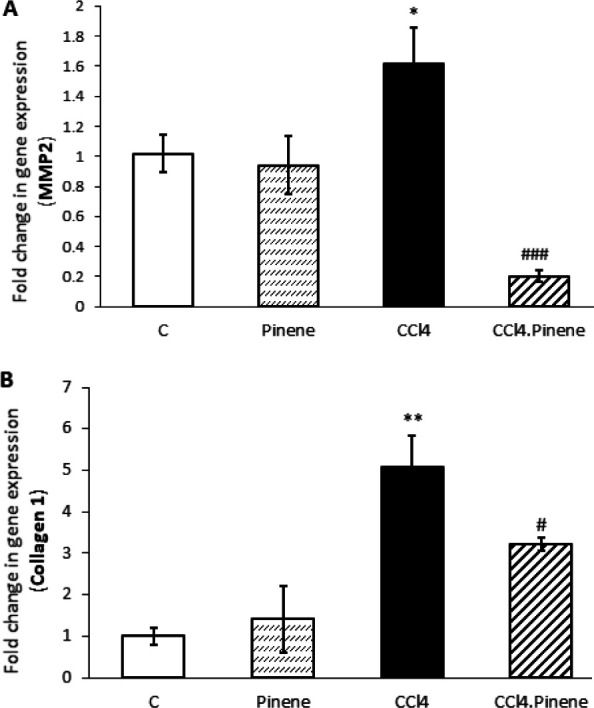
Effect of alpha-pinene on (A) MMP2 and (B) collagen-1 mRNA expression in the liver of CCl_4_-injected rats

We found that rats receiving CCl_4_ had a significant increase in the expression of MMP-2 and Col-1 in their liver compared to the Control group (*P*=0.036 and *P*=0.001, respectively). However, treatment with alpha-pinene led to a reduction in the expression of these genes in the CCl_4_.Pinene group compared to the CCl_4_ group (*P*=0.0001 and *P*=0.049, respectively). The expression of MMP-2 and Col-1 was not significantly different between the Pinene and Control groups, as shown in Figure 4.

### Effect of alpha-pinene on histological alterations in the liver

**Figure 5:**
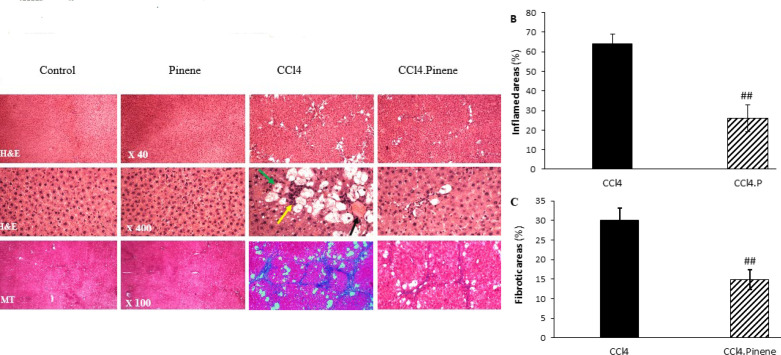
Effect of alpha-pinene on histopathological changes in the liver of CCl_4_-injected rats

Histological examination of liver tissue was conducted after H&E and Masson’s trichrome stainings. Both the control and Pinene groups exhibited a normal hepatic architecture. However, a six-week intraperitoneal administration of CCl_4 _resulted in extensive changes in liver tissue, such as the formation of Mallory-Denk bodies, inflammatory cell infiltration, ballooning degeneration, and steatosis. The liver’s blue coloration due to collagen deposition was detected by Masson’s trichrome staining. In animals that received CCl_4_, alpha-pinene prevented liver tissue destruction (Figure 5A). In addition, the CCl_4_.Pinene group showed a significant reduction in the percentages of inflamed and fibrotic areas compared to the CCl_4_ group (*P*=0.002 and *P*=0.004, respectively) (Figure 5B, C).

### Effect of alpha-pinene on TLR4/NF-κB, PI3K/Akt/mTOR, and TGF-β/Smad2/3 signaling pathways

**Figure 6:**
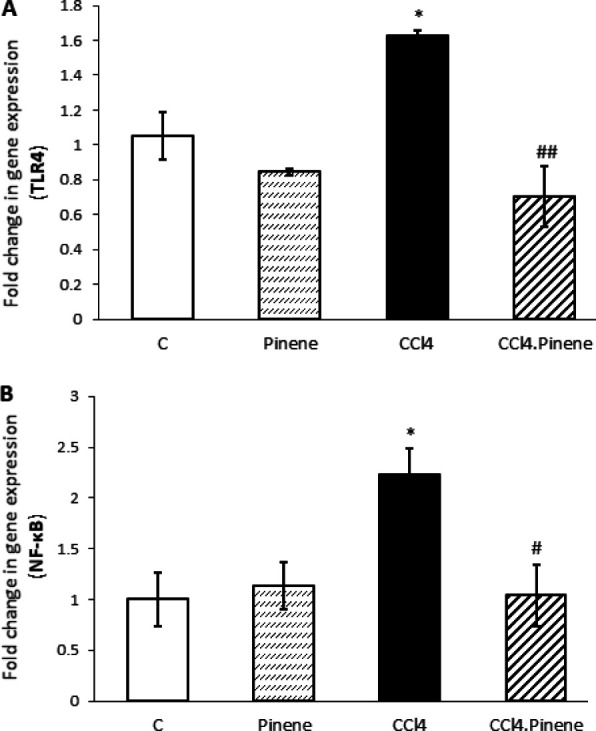
Effect of alpha-pinene on (A) TLR4 and (B) NF-κB mRNA expression in the liver of CCl4-injected rats

Exposure to CCl_4_ caused an increase in the expression of TLR4 and NF-κB in the liver of rats, with statistical significance (*P*=0.028 and *P*=0.011, respectively). However, the expression of these genes was significantly lower in the CCl_4_.Pinene group compared to the CCl_4_ group (*P*=0.004 and *P*=0.013, respectively). The Pinene group did not display any significant difference in the expression of these genes compared to the Control group (Figure 6).

**Figure 7:**
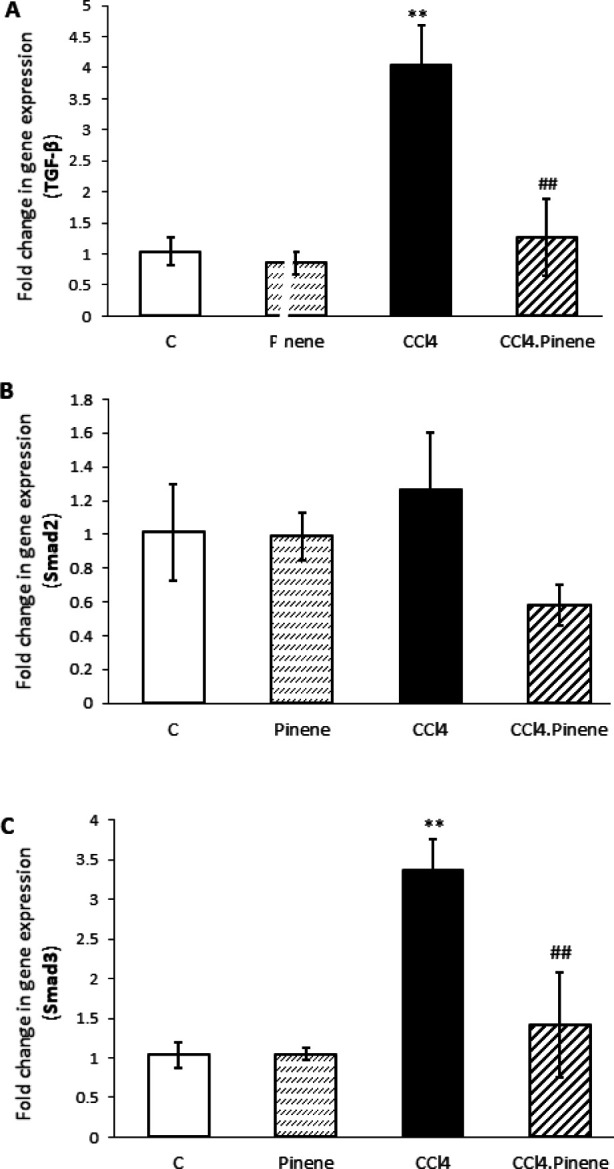
Effect of alpha-pinene on (A) TGF-β, (B) Smad2, and (C) Smad3 mRNA expression in the liver of CCl4-injected rats

The mRNA expression of TGF-β (*P*=0.002) and Smad3 (*P*=0.003) in the liver of rats was significantly increased due to CCl_4_ exposure compared to the Control group. However, there was no significant effect on the expression of Smad2. Treatment with alpha-pinene prevented the increase in the expression of TGF-β and Smad3 in the CCl_4_-exposed group. As a result, the expression of these factors was significantly lower in the CCl_4_.Pinene group compared to the CCl_4_ group (*P*=0.003 and *P*=0.008, respectively). The expression of these genes in the alpha-pinene-treated group was not significantly different from the Control group (Figure 7).

**Figure 8:**
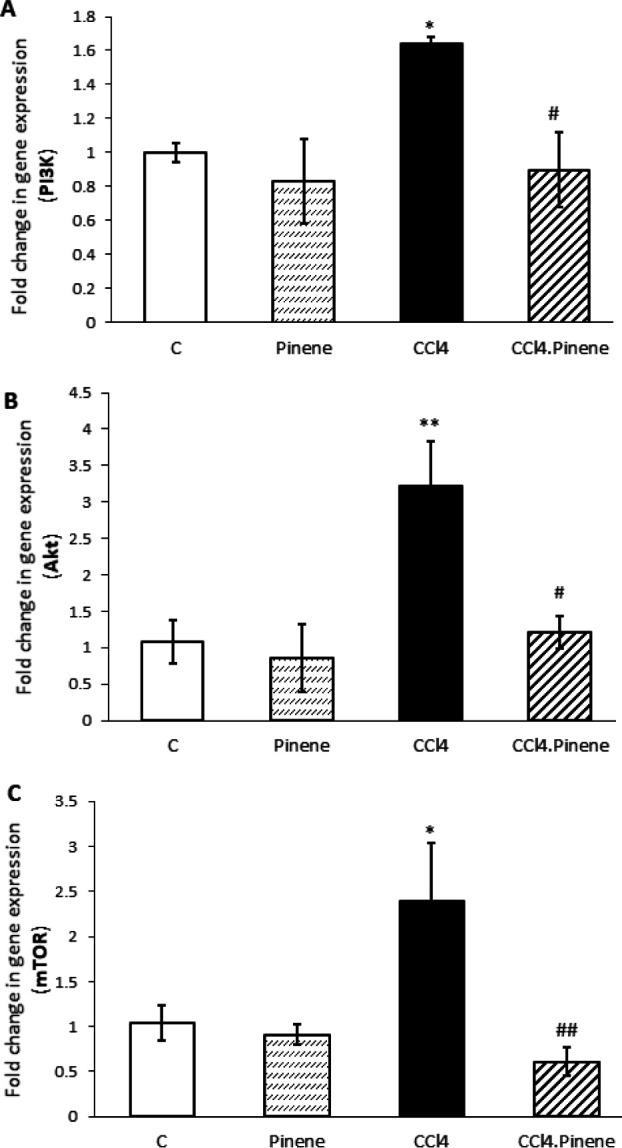
Effect of alpha-pinene on (A) PI3K, (B) Akt, and (C) mTOR mRNA expression in the liver of CCl4-injected rats

The study found that the expression of PI3K (*P*=0.041), Akt (*P*=0.008), and mTOR (*P*=0.028) was significantly higher in the CCl_4_ group than in the Control group. However, treatment with alpha-pinene caused a significant decrease in the expression of these factors in the CCl_4_.Pinene group compared to the CCl_4_ group (*P*=0.024, *P*=0.010, and *P*=0.008, respectively). The Pinene group did not show any significant difference from the Control group (Figure 8).

## Discussion

Injection of CCl_4_ into rats’ peritoneum increased the liver enzyme levels in their serum. It also caused changes in biochemical markers associated with oxidative and nitrosative stress, such as an increase in MDA and NO levels, a decline in GSH levels, and a decrease in catalase activity. Additionally, CCl_4_ increased the expression of proinflammatory factors TNF-α, IL-1β, and IL-6. Moreover, it caused molecular alterations such as increased expression of MMP2 and Col-1 and histological changes related to fibrosis in the liver. Therefore, CCl_4_ injection is a suitable method for generating a fibrosis model in rats’ liver, as shown in previous studies (14, 15, 30).

To investigate the potential of alpha-pinene in inhibiting CCl_4_-induced fibrosis, we administered this monoterpene intraperitoneally at a dosage of 50 mg/kg for six weeks, which corresponds to the duration of CCl_4_ treatment. Notably, alpha-pinene can accumulate significantly in the liver (31), which supports our hypothesis regarding its potential effectiveness.

After injecting alpha-pinene, liver enzyme levels decreased in animals exposed to CCl_4_. In diabetic rats, Santos *et al.* (21) revealed that AST and ALT levels decreased after five consecutive days of alpha-pinene treatment. Previous studies have consistently shown that liver damage caused by CCl_4_ leads to increased liver enzyme levels due to damage to the cell membranes (15, 32, 33), which can result in enzyme and lipid leaks into the bloodstream. It can cause oxidative and nitrosative stress, as well as inflammatory changes, and is a significant symptom of liver disease (1). Therefore, the results of this study suggest that alpha-pinene can help maintain the integrity of liver cell membranes against CCl_4_.

Here, we found that animals in group CCl_4_.Pinene had decreased levels of MDA and NO but higher levels of GSH and catalase in the liver compared to those treated with CCl_4_. Studies show that alpha-pinene has anti-oxidant properties that are effective both in vitro and in vivo (19, 34). The activation of hepatic stellate cells initiates the process of liver fibrosis. Oxidative stress is caused by damaged hepatocytes and activated Kupffer cells, leading to the activation of hepatic stellate cells (2, 35). Oxidative/nitrosative stress induces inflammation and profibrogenic mediators in the liver. For example, inhibiting the production of TGF-β by inhibiting inducible nitric oxide synthase (iNOS) prevents the progression of liver fibrosis. Pharmacological inhibition of iNOS prevents the progression of liver fibrosis (36, 37), whereas alpha-pinene inhibits the expression of iNOS and reduces NO production (38). Our study also shows that alpha-pinene prevents glutathione depletion in liver fibrosis model animals. The reduction of glutathione increases the activity of iNOS in hepatocytes (39). Therefore, the ability of alpha-pinene to inhibit oxidative and nitrosative stresses is the primary factor behind its effectiveness in reducing liver fibrosis.

We observed a significant decrease in the expression of proinflammatory cytokines in the CCl_4_.Pinene group compared to the CCl_4_ group. It is important to note that when the Kupffer cell releases inflammatory mediators, its activity increases, damages hepatocytes, and stimulates hepatic stellate cells. It ultimately leads to the accumulation and deposition of fibrogenic factors (40). The activation of signal transducer and transcription factor 3 (STAT3) by IL-6 also correlates with liver fibrosis and hepatic stellate cell activation (41). Recent studies have shown that *Pinus mugo* essential oil, which contains significant levels of alpha-pinene, inhibits STAT3 phosphorylation and activation (42). Since alpha-pinene reduced the expression of IL-6 in the livers of rats injected with CCl_4_, its anti-fibrogenic effects may be partially mediated by suppressing this signaling pathway. Therefore, we suggest investigating the STAT3 phosphorylation in the liver of CCl_4_.Pinene animals. Furthermore, the down-regulation of IL-1β expression in the liver of CCl_4_-injected rats may contribute to alpha-pinene’s anti-fibrotic effects, as IL-1β and its receptor stimulate fibrogenesis in a CCl_4_-induced model (43). 

The results revealed that alpha-pinene can prevent the increase in NF-B expression in the liver of rats injected with CCl_4_. It is worth noting that proinflammatory factors such as TNF-α, IL-6, and IL-1β are downstream of NF-κB, and activation of this transcription factor leads to increased expression of proinflammatory cytokines (44). A previous study by Kang et al. (45) showed that alpha-pinene represses TNF-α signaling by down-regulating NF-κB in MDA-MB-231 human breast cancer cells and acts as an inhibitor of tumor invasion, which is consistent with the findings of our study. It is important to note that TLR4/NF-κB signaling strengthens the fibrogenic pathway of TGF-β (46), and its suppression is considered a therapeutic target in liver fibrosis (13, 25, 47). Our study found that alpha-pinene reduced the mRNA expression of TLR4 and NF-κB in rats receiving CCl_4_. However, we suggest further investigation of the expression of the proteins of this pathway.

Also, we found that alpha-pinene reduced MMP2 expression in the liver of rats injected with CCl_4_, which is similar to the findings of Karthikeyan et al. (48). They observed that alpha-pinene inhibited MMP2 expression in the skin of UVA-irradiated mice. TGF-β triggers the activation of hepatic stellate cells and increases the expression of MMP2, which in turn induces matrix contraction. This process leads to an imbalance between matrix production and destruction, resulting in fibrosis (2, 49). Therefore, the ability of alpha-pinene to reduce MMP2 expression depends on its ability to inhibit the increase in TGF-β.

In the current study, CCl_4_ increased the expression of TGF-β and Smad3 mRNA in the liver. However, it has no significant effect on Smad2 expression. Smads are intracellular effectors of TGF-β. When Smad2 is overexpressed, it reduces collagen deposition in the liver. Conversely, overexpression of Smad3 leads to increased expression of collagen type 1 and proinflammatory cytokines and activation of hepatic stellate cells. Therefore, Smad3 plays a crucial role in liver fibrosis in response to TGF-β. As a result, TGF-β/Smad2/3 signaling has been considered a therapeutic target for fibrosis (50, 51). Our observations show that alpha-pinene reduced the expression of both TGF-β and Smad3 in animals receiving CCl_4_. This reduction was associated with decreased Col-1 mRNA expression and reduced collagen deposition in the liver of CCl_4_.Pinene animals. Ko *et al.* (52) also found that pycnogenol (pine bark extract) reduces the expression of TGF-β and decreases the phosphorylation of Smad3. Since a large amount of alpha-pinene can be found in pycnogenol (53), suppressing the TGF-β/Smad3 signaling pathway may have contributed to the anti-fibrotic effect of alpha-pinene. Therefore, it is necessary to investigate the activity of Smad3 in the CCl_4_.Pinene group.

In the hepatic stellate cells, activating the PI3K/Akt/mTOR signaling pathway links to liver damage, such as fibrosis and cancer (54). This pathway is non-Smad and activated by TGF-β (55). Since a high expression of mTOR worsens CCl_4_-induced fibrosis, mTOR is considered one of TGF-β’s partners in inducing liver fibrosis (56, 57). Additionally, inhibiting the PI3K/Akt/mTOR signaling pathway helps prevent collagen type 1 protein production and TGF-β transcription and translation (58). By studying mRNA levels of factors involved in this signaling pathway, we observed that alpha-pinene prevents fibrosis progression in animals treated with CCl_4_ by inhibiting this defective cycle.

In addition, oxidative and nitrosative stress contribute to collagen production in fibrosing liver diseases (59). Considering the ability of alpha-pinene to hinder the oxidative damage caused by CCl_4_ in the liver, the reduction of collagen production and deposition in the liver (proved by Masson’s trichrome staining) was also predictable. 

Moreover, alpha-pinene partially prevented multi-histopathological alterations in the hepatic tissue of animals receiving CCl_4_. This compound also protects against tissue changes in acute pancreatitis, and these outcomes have been attributed to its anti-oxidant, anti-inflammatory, and anti-apoptotic properties (60). The alpha-pinene dosage in this study was 50 mg/kg for six weeks at the same time as the CCl_4_ treatment. Higher doses or longer treatment durations of this monoterpene may result in complete prevention of liver damage.

**Table 1:** The primer sequences for the relevant genes were utilized in the real-time PCR

Gene	Forward primers sequence (5'–3')	Reverse primers sequence (5'–3')
TNF-α	CACGGGAGCCGTGACTGTA	TCCAAGCGAACTTTATTTCTCTCA
IL-1β	TCAGGAAGGCAGTGTCACTCA	TCCACGGGCAAGACATAGGT
IL-6	ACTATGAGGTCTACTCGGCAAACC	ACAGTGAGGAATGTCCACAAACTG
NF-κB	CATGGCAGACGACGATCCTT	TGGAGTGAGTCAAAGCAGTATTCAA
TLR4	AGCCTTGAATCCAGATGAAAC	ACAGCAGAAACCCAGATGAA
Col-1	AGCTTCACCCTTAGCACCAG	GTGGTAACGATGGTGCTGTC
MMP2	AGACAAAGAGTTGGCAGTGCAAT	CTGTATGTGATCTGGTTCTTGTCCC
TGF-B	TGCTTCAGCTCCACAGAGAA	TGGTTGTAGAGGGCAAGGAC
Smad2	GTGTTTGCCGAGTGCCTAAGT	TTACAGCCTGGTGGGATTTTG
Smad3	GGACGCAGGCTCTCCAAAC	AGGAGATGGAGCACCAAAAGG
PI3K	GACAGGCACAACGACAAC	AAGCCCTAACGCAGACAT
Akt	GCTCTTCTTCCACCTGTCTCG	CACAGCCCGAAGTCCGTTA
mTOR	CTGATGTCATTTATTGGCACAAA	CAGGGACTCAGAACACAAATGC
GAPDH	GCTACACTGAGGACCAGGTTGTCT	CCCAGCATCAAAGGTGGAA

**Table 2:** Effect of alpha-pinene on plasma levels of aspartate aminotransferase (AST), alanine aminotransferase (ALT), alkaline phosphatase (ALP), and gamma-glutamyl transferase (GGT) in CCl_4_-injected rats

GGT (U/ml)	ALP (U/ml)	ALT (U/ml)	AST (U/ml)	Group
1.72 ± 0.04	76.49 ± 1.87	55.34 ± 2.32	93.25 ± 1.11	Control
2.11 ± 0.19	77.00 ± 4.91	45.95 ± 0.55	87.40 ± 3.58	Pinene
3.51 ± 0.22 ***	255.14 ± 10.22 ***	84.01 ± 0.67 ***	423.83 ± 4.26 ***	CCl4
2.11 ± 0.25 ###	221.23 ± 2.43 ##	56.36 ± 9.24 ###	249.11 ± 3.39 ###	CCl4.Pinene

Results are presented as means ± SEM. Each group contained six rats, and each sample was measured three times. *P<0.05, **P<0.01, and ***P<0.001 vs Control group and #*P*<0.05 and ##*P*<0.01 vs CCl4 group.

Data are presented as mean ± SEM; n = 5. *P<0.05, **P<0.01 vs Control group and #*P*<0.05, ##*P*<0.01 vs CCl_4_ group.

Data are presented as mean ± SEM; n = 5. *P<0.05, **P<0.01 vs Control group and #*P*<0.05, ###*P*<0.001 vs CCl4 group.

(A) Photomicrographs were prepared from liver sections stained with hematoxylin and eosin (H&E) and Masson's trichrome (MT). The infiltration of inflammatory cells (yellow arrow), ballooning degeneration (green arrow), and Mallory-Denk bodies (black arrow) are frequently observed in the liver sections of the CCl4 group stained with H&E. The effect of alpha-pinene on the percentage of (B) inflamed areas and (C) fibrotic areas in rats receiving CCl4. Five animals from each group and ten fields from each animal were examined microscopically. Data are presented as mean ± SEM. ##*P*<0.01 vs CCl4 group.

Data are presented as mean ± SEM; n = 5. *P<0.05 vs Control group and #*P*<0.05, ##*P*<0.01 vs CCl_4_ group.

Data are presented as mean ± SEM; n = 5. **P<0.01 vs Control group and ##*P*<0.01 vs CCl_4_ group.

Data are presented as mean ± SEM; n = 5. *P<0.05, **P<0.01 vs Control group and #*P*<0.05, ##*P*<0.01 vs CCl_4_ group.

## Conclusion

Overall, we concluded that alpha-pinene has a beneficial effect on the liver. It prevents oxidative damage and inflammation, which, in turn, helps prevent the development and progression of fibrosis caused by CCl_4_. This monoterpene achieves its effect by inhibiting the TLR4/NF-κB, TGF-β/Smad3, and PI3K/Akt/mTOR signaling pathways. Consequently, alpha-pinene may have potential therapeutic value in treating liver diseases.

## Declaration

Grammarly is used to improve language and readability.After using this tool, the author(s) reviewed and edited the content as needed and take full responsibility for the content of the publication.

## References

[1] Unsal V, Cicek M, Sabancilar İ (2021). Toxicity of carbon tetrachloride, free radicals and role of anti-oxidants. Environ Health Rev.

[2] Weber LW, Boll M, Stampfl A (2003). Hepatotoxicity and mechanism of action of haloalkanes: Carbon tetrachloride as a toxicological model. Crit Rev Toxicol.

[3] Zhu W, Fung PC (2000). The roles played by crucial free radicals like lipid free radicals, nitric oxide, and enzymes NOS and NADPH in CCl4-induced acute liver injury of mice. Free Radic Biol Med.

[4] Beddowes EJ, Faux SP, Chipman JK (2003). Chloroform, carbon tetrachloride and glutathione depletion induce secondary genotoxicity in liver cells via oxidative stress. Toxicology.

[5] Simeonova PP, Gallucci RM, Hulderman T, Wilson R, Kommineni C, Rao M (2001). The role of tumor necrosis factor-α in liver toxicity, inflammation, and fibrosis induced by carbon tetrachloride. Toxicol Appl Pharmacol.

[6] Monserrat-Mesquida M, Quetglas-Llabrés M, Abbate M, Montemayor S, Mascaró CM, Casares M (2020). Oxidative stress and proinflammatory status in patients with nonalcoholic fatty liver disease. Antioxidants.

[7] Zhang G, Wang X, Chung TY, Ye W, Hodge L, Zhang L (2020). Carbon tetrachloride (CCl4) accelerated development of nonalcoholic fatty liver disease (NAFLD)/steatohepatitis (NASH) in MS-NASH mice fed western diet supplemented with fructose (WDF). BMC Gastroenterol.

[8] Pei Q, Yi Q, Tang L (2023). Liver fibrosis resolution: from molecular mechanisms to therapeutic opportunities. Intl J Mol Sci.

[9] Hu HH, Chen DQ, Wang YN, Feng YL, Cao G, Vaziri ND (2018). New insights into TGF-β/Smad signaling in tissue fibrosis. Chem Biol Interact.

[10] Hayashi H, Sakai T (2012). Biological significance of local TGF-β activation in liver diseases. Front Physiol.

[11] Kurzepa J, Mdro A, Czechowska G, Kurzepa J, Celiński K, Kazmierak W (2014). Role of MMP-2 and MMP-9 and their natural inhibitors in liver fibrosis, chronic pancreatitis and non-specific inflammatory bowel diseases. Hepatobiliary Pancreat Dis Int.

[12] Wei L, Chen Q, Guo A, Fan J, Wang R, Zhang H (2018). Asiatic acid attenuates CCl4-induced liver fibrosis in rats by regulating the PI3K/AKT/mTOR and Bcl-2/Bax signaling pathways. Int Immunopharmacol.

[13] Dong Z, Zhuang Q, Ning M, Wu S, Lu L, Wan X (2020). Palmitic acid stimulates NLRP3 inflammasome activation through TLR4-NF-κB signal pathway in hepatic stellate cells. Ann Transl Med.

[14] Ogaly HA, Aldulmani SA, Al-Zahrani FA, Abd-Elsalam RM (2022). D-carvone attenuates CCl4-induced liver fibrosis in rats by inhibiting oxidative stress and TGF-ß1/SMAD3 signaling pathway. Biology.

[15] Abdelghffar EA, Obaid WA, Alamoudi MO, Mohammedsaleh ZM, Annaz H, Abdelfattah MA (2022). Thymus fontanesii attenuates CCl4-induced oxidative stress and inflammation in mild liver fibrosis. Biomed Pharmacother.

[16] Wang T, Lu Z, Sun GF, He KY, Chen ZP, Qu XH (2024). Natural products in liver fibrosis management: A five-year review. Curr Med Chem.

[17] Allenspach M, Steuer C (2021). α-Pinene: A never-ending story. Phytochemistry.

[18] Zhang B, Wang H, Yang Z, Cao M, Wang K, Wang G (2020). Protective effect of alpha-pinene against isoproterenol-induced myocardial infarction through NF-κB signaling pathway. Hum Exp Toxicol.

[19] Xanthis V, Fitsiou E, Voulgaridou GP, Bogadakis A, Chlichlia K, Galanis A (2021). Anti-oxidant and cytoprotective potential of the essential oil Pistacia lentiscus var chia and its major components myrcene and α-pinene. Antioxidants.

[20] Khan-Mohammadi-Khorrami MK, Asle-Rousta M, Rahnema M, Amini R (2022). Neuroprotective effect of alpha-pinene is mediated by suppression of the TNF-α/NF-κB pathway in Alzheimer’s disease rat model. J Biochem Mol Toxicol.

[21] Santos ES, de Sousa Machado ST, Rodrigues FB, da Silva YA, Matias LC, Lopes MJ (2023). Potential anti-inflammatory, hypoglycemic, and hypolipidemic activities of alpha-pinene in diabetic rats. Process Biochem.

[22] Noroozi F, Asle-Rousta M, Amini R, Sahraeian Z (2024). Alpha-pinene alleviates CCl4-induced renal and testicular injury in rats by targeting oxidative stress, inflammation, and apoptosis. Iran J Basic Med Sci.

[23] Hsouna AB, Dhibi S, Dhifi W, Mnif W, Hfaiedh N (2019). Chemical composition and hepatoprotective effect of essential oil from Myrtus communis L flowers against CCL4-induced acute hepatotoxicity in rats. RSC Adv.

[24] Mohamed ME, Younis NS, El-Beltagi HS, Mohafez OM (2022). The synergistic hepatoprotective activity of rosemary essential oil and curcumin: The role of the MEK/ERK pathway. Molecules.

[25] Wang K, Yang X, Wu Z, Wang H, Li Q, Mei H (2020). Dendrobium officinale polysaccharide protected CCl4-induced liver fibrosis through intestinal homeostasis and the LPS-TLR4-NF-κB signaling pathway. Front Pharmacol.

[26] ElBaset MA, Salem RS, Ayman F, Ayman N, Shaban N, Afifi SM (2022). Effect of empagliflozin on thioacetamide-induced liver injury in rats: role of AMPK/SIRT-1/HIF-1α pathway in halting liver fibrosis. Antioxidants.

[27] Lowry OH, Rosebrough NJ, Farr AL, Randall RJ (1951). Protein measurement with the Folin phenol reagent. J Biol Chem.

[28] Draper HH, Hadley M (1990). Malondialdehyde determination as index of lipid Peroxidation. Methods Enzymol.

[29] Livak KJ, Schmittgen TD (2001). Analysis of relative gene expression data using real-time quantitative PCR and the 2−ΔΔCT method. Methods.

[30] Wang Y, Yang Z, Wei Y, Li X, Li S (2021). Apolipoprotein A4 regulates the immune response in carbon tetrachloride-induced chronic liver injury in mice. Int Immunopharmacol.

[31] Satou T, Kasuya H, Maeda K, Koike K (2014). Daily inhalation of α-pinene in mice: Effects on behavior and organ accumulation. Phytother Res.

[32] Eidi A, Mortazavi P, Moghadam JZ, Mardani PM (2015). Hepatoprotective effects of Portulaca oleracea extract against CCl4-induced damage in rats. Pharm Biol.

[33] Alamri ES, El Rabey HA, Alzahrani OR, Almutairi FM, Attia ES, Bayomy HM (2022). Enhancement of the protective activity of vanillic acid against tetrachloro-carbon (CCl4) hepatotoxicity in male rats by the synthesis of silver nanoparticles (AgNPs). Molecules.

[34] Bouzenna H, Hfaiedh N, Giroux-Metges MA, Elfeki A, Talarmin H (2017). Potential protective effects of alpha-pinene against cytotoxicity caused by aspirin in the IEC-6 cells. Biomed Pharmacother.

[35] Svegliati Baroni G, D’Ambrosio L, Ferretti G, Casini A, Di Sario A, Salzano R (1998). Fibrogenic effect of oxidative stress on rat hepatic stellate cells. Hepatology.

[36] Sánchez-Valle V, C Chavez-Tapia N, Uribe M, Méndez-Sánchez N (2012). Role of oxidative stress and molecular changes in liver fibrosis: A review. Curr Med Chem.

[37] Iwakiri Y (2015). Nitric oxide in liver fibrosis: The role of inducible nitric oxide synthase. Clin Mol Hepatol.

[38] Kim DS, Lee HJ, Jeon YD, Han YH, Kee JY, Kim HJ (2015). Alpha-pinene exhibits anti-inflammatory activity through the suppression of MAPKs and the NF-κB pathway in mouse peritoneal macrophages. Am J Chinese Med.

[39] Harbrecht BG, Di Silvio M, Chough V, Kim YM, Simmons RL, Billiar TR (1997). Glutathione regulates nitric oxide synthase in cultured hepatocytes. Ann Surg.

[40] Ignat SR, Dinescu S, Hermenean A, Costache M (2020). Cellular interplay as a consequence of inflammatory signals leading to liver fibrosis development. Cells.

[41] Xiang DM, Sun W, Ning BF, Zhou TF, Li XF, Zhong W (2018). The HLF/IL-6/STAT3 feedforward circuit drives hepatic stellate cell activation to promote liver fibrosis. Gut.

[42] Thalappil MA, Butturini E, Carcereri de Prati A, Bettin I, Antonini L, Sapienza FU (2022). Pinus mugo essential oil impairs STAT3 activation through oxidative stress and induces apoptosis in prostate cancer cells. Molecules.

[43] Meier RP, Meyer J, Montanari E, Lacotte S, Balaphas A, Muller YD (2019). Interleukin-1 receptor antagonist modulates liver inflammation and fibrosis in mice in a model-dependent manner. Int J Mol Sci.

[44] Liu T, Zhang L, Joo D, Sun SC (2017). NF-κB signaling in inflammation. Signal Transduct Target Ther.

[45] Kang E, Lee DH, Jung YJ, Shin SY, Koh D, Lee YH (2016). α-Pinene inhibits tumor invasion through down-regulation of nuclear factor (NF)-κB-regulated matrix metalloproteinase-9 gene expression in MDA-MB-231 human breast cancer cells. Appl Biol Chem.

[46] Seki E, De Minicis S, Österreicher CH, Kluwe J, Osawa Y, Brenner DA (2007). TLR4 enhances TGF-β signaling and hepatic fibrosis. Nature Med.

[47] Zhao D, Xue C, Yang Y, Li J, Wang X, Chen Y (2022). Lack of Nogo-B expression ameliorates PPARγ deficiency-aggravated liver fibrosis by regulating TLR4-NF-κB-TNF-α axis and macrophage polarization. Biomed Pharmacother.

[48] Karthikeyan R, Kanimozhi G, Madahavan NR, Agilan B, Ganesan M, Prasad NR (2019). Alpha-pinene attenuates UVA-induced photoaging through inhibition of matrix metalloproteinases expression in mouse skin. Life Sci.

[49] Eldred JA, Hodgkinson LM, Dawes LJ, Reddan JR, Edwards DR, Wormstone IM (2012). MMP2 activity is critical for TGFβ2-induced matrix contraction—Implications for fibrosis. Investig Ophthalmol Vis Sci.

[50] Xu F, Liu C, Zhou D, Zhang L (2016). TGF-β/SMAD pathway and its regulation in hepatic fibrosis. J Histochem Cytochem.

[51] Zhangdi HJ, Su SB, Wang F, Liang ZY, Yan YD, Qin SY (2019). Crosstalk network among multiple inflammatory mediators in liver fibrosis. World J Gastroenterol.

[52] Ko JW, Shin NR, Park SH, Kim JS, Cho YK, Kim JC (2017). Pine bark extract (Pycnogenol®) suppresses cigarette smoke-induced fibrotic response via transforming growth factor-β1/Smad family member 2/3 signaling. Lab Anim Res.

[53] Ustun O, Senol FS, Kurkcuoglu M, Orhan IE, Kartal M, Baser KH (2012). Investigation on chemical composition, anticholinesterase and anti-oxidant activities of extracts and essential oils of Turkish Pinus species and pycnogenol. Ind Crops Prod.

[54] Golob-Schwarzl N, Krassnig S, Toeglhofer AM, Park YN, Gogg-Kamerer M, Vierlinger K (2017). New liver cancer biomarkers: PI3K/AKT/mTOR pathway members and eukaryotic translation initiation factors. Eur J Cancer.

[55] Zhang YE (2009). Non-Smad pathways in TGF-β signaling. Cell Res.

[56] Shan L, Ding Y, Fu Y, Zhou L, Dong X, Chen S (2016). mTOR overactivation in mesenchymal cells aggravates CCl4- induced liver fibrosis. Sci Rep.

[57] Jiménez-Uribe AP, Gómez-Sierra T, Aparicio-Trejo OE, Orozco-Ibarra M, Pedraza-Chaverri J (2021). Backstage players of fibrosis: NOX4, mTOR, HDAC, and S1P; companions of TGF-β. Cell Signal.

[58] Magaye RR, Savira F, Hua Y, Xiong X, Huang L, Reid C (2021). Attenuating PI3K/Akt-mTOR pathway reduces dihydrosphingosine 1 phosphate mediated collagen synthesis and hypertrophy in primary cardiac cells. Int J Biochem Cell Biol.

[59] Antar SA, Ashour NA, Marawan ME, Al-Karmalawy AA (2023). Fibrosis: Types, effects, markers, mechanisms for disease progression, and its relation with oxidative stress, immunity, and inflammation. Int J Mol Sci.

[60] Bae GS, Park KC, Choi SB, Jo IJ, Choi MO, Hong SH, Song K, Song HJ, Park SJ (2012). Protective effects of alpha-pinene in mice with cerulein-induced acute pancreatitis. Life Sci.

